# A Case of Severe Nervous Irritation, from the Sting of the Insect Called Yellow Jacket, [*Vespa Crabro*,] Communicated in a Letter from Dr. S. Littel, Jr., to Dr. Drake

**Published:** 1830

**Authors:** S. Littell


Art.—V. *A case of severe Nervous Irritation, from the sting of the insect called a Yellow Jacket [Vespa Crabro] in a letter from* Dr. Littell to Dr. Drake.

Dear Sir,—

I am aware that cases of extraordinary nature, and of rare occurrence, are generally of little practical utility, but the following one, which came under my notice a few days since, seems to me sufficiently interesting to merit preservation; should it also appear so to you, I would be gratified by its insertion in your journal.

Charles Jacoby, a German, about 40 years of age, of vigorous health and robust constitution, while ploughing on the sixth of August, was stung about the middle of the fore arm by a *yellow jacket*, [Vespa Crabro] the pain was not greater than is usual in such cases, but was almost immediately followed by a slight chill, succeeded by a flash of heat ; after the lapse of about ten minutes, an *aura*, which he described as compounded of heat and a sharp prickling sensation, was felt in the wound, and gradually diffused itself over the whole body ; after this had continued a short time, vision became indistinct, and soon failed altogether ; he called to his companion, who was about a hundred yards distant, and conversed with him two or three minutes after his arrival, when he suddenly fell from his seat in a state of total insensibility, in which he continued nearly two hours ; his limbs were rigidly extended, his mouth was firmly clasped and slightly covered with foam ; there seemed to be a complete cessation of the functions of the heart and lungs, for those around him stated that during the space of half an hour, neither respiration nor pulsation was perceptible, his eyes were fixed and ghastly, face livid, almost black ; his fingers also, assumed a bluish hue, and his extremities became quite cold. In this condition he continued about thirty minutes without any evident change. At the expiration of this time, some respiratory movements were perceptible, feeble and irregular at first, but gradually becoming stronger, with a corresponding improvement in the pulse, which was also feeble and intermitting on its reappearance ; the livid colour forsook his lips and the amendment though gradual, was progressive, till I saw him, about two hours from the commencement of the symptoms. I spake to him, shortly after I entered the house, and he replied correctly, complaining of pain over his whole body ; and a little while afterwards, while I was revolving in my mind the nature of his malady, (for at this time I was not aware that he had been stung) he opened his eyes, sat up in bed, and seemed to be restored to the full possession of his faculties ; for he now recognized those around him. He was utterly unconscious of all that had transpired since the accession of the blindness, not even remembering the short conversation he had with his friend ; whose approach towards him after he had been called, was the last thing which he could recollect. Though still somewhat dull and drowsy, and a little hoarse, he conversed freely, pointed out the spot where he had been stung, &c. His respiration was now easy and natural, pulse weak but regular, and warmth was gradually diffusing itself over the extremities, which however were still cool ; the pupils of his eyes were unaffected, he complained of severe pain in the head and of chilliness, for which I directed a warm pedilurium with salt, and hot brandy toddy, the only appropriate remedies at hand—these measures afforded him some relief, but, at the time I write, he states that he still feels the effects of his illness. Mr. Jacoby informs me that he had a similar attack about four years ago, and that, to the best of his recollection, he was stung by a *Yellow Jacket* on that occasion also, though his symptoms were not then referred to that cause. He has been frequently stung by bees, wasps, &c. without suffering any unusual consequence.

Very respectfully,

S. LITTELL, jr.

